# The Urine Light Chain/eGFR Quotient as a Tool to Rule out Cast Nephropathy in Myeloma-Associated Kidney Failure

**DOI:** 10.3390/biomedicines12051032

**Published:** 2024-05-08

**Authors:** David Klank, Christian Löffler, Julian Friedrich, Martin Hoffmann, Peter Paschka, Raoul Bergner

**Affiliations:** 1 Medizinische Klinik A, Klinikum der Stadt Ludwigshafen gGmbH, Bremserstraße 79, 67063 Ludwigshafen, Germany; julian.friedrich@medma.uni-heidelberg.de (J.F.); hoffmanm@klilu.de (M.H.); paschkap@klilu.de (P.P.);; 2 Klinik für Innere Medizin, Rheumatologie und Immunologie, Medius Klinik Kirchheim, Eugenstr. 3, 73230 Kirchheim unter Teck, Germany; c.loeffler2@medius-kliniken.de; 3 Department of Nephrology, Endocrinology, Hypertensiology and Rheumatology, University Hospital Mannheim, University of Heidelberg, 69117 Heidelberg, Germany

**Keywords:** multiple myeloma, kidney biopsy, cast nephropathy

## Abstract

Kidney involvement with resulting kidney failure leads to increased mortality in patients with multiple myeloma (MM). Cast nephropathy (CN), in particular, if left untreated, quickly leads to kidney failure requiring dialysis and has a very poor prognosis for the affected patient. The gold standard for diagnosing kidney involvement is a kidney biopsy. However, due to bleeding risk, this cannot be done in every patient. We recently reported that a quotient of urine light chain (LCurine) to glomerular filtration rate (eGFR) is a non-invasive diagnostic tool for patients with kidney involvement in MM. But this quotient has not yet been tested in everyday clinical practice. In this study, our LCurine/eGFR ratio was tested on 67 patients in two centers. Enrollment took place between January 2019 and September 2023. A total of 18 of the 67 patients had CN. With the threshold defined in our initial paper, we were able to show a sensitivity of 100% with a specificity of 85.7% for CN in patients with MM. As a result, the LCurine/eGFR quotient recognizes 100% of all CN and can therefore detect this group, which has a very poor prognosis, without the need for a kidney biopsy.

## 1. Introduction

Previously, we reported for the first time on a non-invasive approach to diagnose myeloma-associated cast nephropathy (CN) using the urine light chain (LCurine)/glomerular filtration rate (eGFR) quotient [1]. About 20–40% of patients with multiple myeloma (MM) develop kidney failure due to light chain (LC) nephropathy, with CN being a common cause [2,3]. The International Myeloma Working Group (IMWG) currently recommends determining serum creatinine, creatinine clearance, estimated glomerular filtration rate (eGFR), electrolyte levels, and blood urea nitrogen to clarify possible kidney involvement by multiple myeloma. But only CN counts as a myeloma-defining event within the framework of the so-called CRAB criteria (C = Hypercalcemia: serum calcium > 0.25 mmol/L (>1 mg/dL) higher than the upper limit of normal or >2.75 mmol/L (>11 mg /dL); R = Renal failure: creatinine clearance < 40 mL per minute or serum creatinine > 177 mol/L (>2 mg/dL); A = Anemia: hemoglobin value of >20 g/L below the lowest limit of normal, or a hemoglobin value < 100 g/L; and B = Bone lesions: one or more osteolytic lesion on skeletal radiography, CT, or PET/CT) [4].

When untreated, CN rapidly progresses to acute kidney failure requiring dialysis, with a poor prognosis for the patients [5,6,7]. Therefore, rapid and effective MM treatment is required to improve kidney function and consecutively the patient’s prognosis. With the rapid use of modern anti-myeloma therapy (the proteasome inhibitor bortezomib has proven particularly useful here), kidney function can be significantly improved in many patients. But it is also a well-known phenomenon that only patients who respond hematologically also show an improving kidney function [8,9].

However, only in about 60% of cases of MM-associated kidney failure is CN the underlaying renal disease [10]. In addition to CN, there is a broad spectrum of histologic findings in the kidneys of patients with paraproteinemia and/or paraproteinuria [10]. Other patients suffer from deposits of light chains, which leads to AL amyloidosis or monoclonal immunoglobulin deposition disease (MIDD), from toxic effects of light chains, as in light chain proximal tubulopathy, or the direct infiltration of the kidney by myeloma cells. However, other kidney diseases independent of paraproteinemia can also occur, such as diabetic nephropathy, primary glomerular disease, or nephroangiosclerosis. The gold standard for diagnosing the different kidney diseases is kidney biopsy. However, in some patients the excess in light chains results in an interaction with thrombocyte aggregation, leading to an increased risk of bleeding, which in turn prevents the performance of a kidney biopsy. There is also a group of patients in whom anticoagulation cannot be stopped due to acute events such as myocardial infarction with stent insertion.

Thus, the quotient recently established by our team offers a diagnostic tool to identify patients with CN without the need for a kidney biopsy. As shown in our first publication, only the LCurine/eGFR quotient has a sufficiently high sensitivity and specificity to detect CN. Neither the LCurine concentration alone nor the eGFR are sufficient. The LCurine/eGFR quotient takes advantage of the fact that CN has a more severe impairment of kidney function than other paraprotein-associated kidney diseases, but with a significantly higher excretion of LC in the urine. For lambda LC, the highest accuracy was calculated at an LCurine/eGFR quotient of >2 (the sensitivity and specificity for CN were 94% and 90%). For kappa LC, it is either a LCurine/eGFR ratio > 1 and proteinuria < 8 g/24 h or a LCu/eGFR > 5 (sensitivity 87% and specificity 81%).

So far, the quotient has never been validated in daily routine. In this study, we sought to validate a non-invasive approach to predicting the presence or absence of CN by using the LCurine/eGFR quotient.

## 2. Materials and Methods

Study design und selection of patients.

We conducted this retrospective observational study in two centers on 67 patients who received a kidney biopsy during their inpatient stay. The aim of the study was to check the LCurine/eGFR quotient we described in everyday clinical practice.

The indication for kidney biopsy was either the presence of a paraprotein and an unclear kidney failure (eGFR < 60 mL/min/1.73 m^2^ or an unclear proteinuria > 500 mg/24 h) or an unclear monoclonal light chain excretion in the urine. The paraprotein could be either an intact immunoglobulin or just a light chain. The kidney biopsy and data collection took place between January 2019 and September 2023. All biopsies were performed in two medical centers. Patients in whom a kidney biopsy could not be performed due to a severe blood clotting disorder or ongoing anticoagulation were excluded from the study. Written informed consent for kidney biopsy was mandatory.

### 2.1. Kidney Biopsy

In both hospitals, kidney biopsies are performed as a standardized procedure and require at least one overnight stay for patients for monitoring. In this way, complications are also recorded and treated. Before the kidney biopsy, coagulation diagnostics are carried out in order to reduce bleeding complications as much as possible. Depending on their mode of action, oral anticoagulants must be stopped a few days beforehand. On the day of the kidney biopsy, strict blood pressure control is carried out. In the event of an increase in blood pressure after the biopsy, patients are monitored for one night in an intermediate care ward.

### 2.2. Data Collection and Laboratory Analyses

The data collected included the following: patient characteristics (age, sex), kidney function (creatinine, eGFR calculated using the CKD-EPI formula), proteinuria (measured as mg per 24 h), LC concentration in urine, and LC type (kappa, lambda). The data were collected at the time of kidney biopsy. For our quotient, we used the limits that we defined in our initial publication. These are for lambda-LC: LCurine/eGFR quotient > 2; for kappa-LC: LCurine/eGFR quotient > 5 or LCurine/eGFR quotient 1–5 and proteinuria < 8 g/day.

HydraGel^®^ kits (Sebia Labordiagnostische Systeme GmbH, Fulda, Germany) were used for immunofixation to detect Bence Jones proteinuria. With the Freelite Assay^®^ (Binding Site GmbH, Schwetzingen, Germany) we quantified serum free LCs, and the urine LCs were quantified nephelometrically with BN ProSpec ^®^ (Siemens Healthcare GmbH, Erlangen, Germany). Proteinuria was quantified turbidimetrically with an automated machine (Architect ci 8200^®^, Abbott GmbH, Wiesbaden, Germany). The CKD-EPI formula was used to calculate the estimated glomerular filtration rate (eGFR). This was in accordance with the International Myeloma Working Group recommendations. If myeloma was first diagnosed through a kidney biopsy or myeloma was suspected, further diagnostics were carried out (bone marrow biopsy, CT and MRI diagnostics, etc.).

The pathological workup included conventional histology on 1 µm thin hematoxylin-eosin and periodic acid–Schiff stains, immunohistochemistry with IgA, IgG, IgM, light chain kappa, light chain lambda, C1q and C3 antibodies, and electron microscopy. Congo-red staining was included in patients who showed proteinuria or depositions suspicious of amyloid. All biopsies were examined by an experienced kidney pathologist.

### 2.3. Statistical Analysis

After kidney histology was available, the results were divided into CN and other kidney disease (OKD). The sensitivity and specificity of our LCurine/eGFR quotient were calculated. For statistical analysis, WINSTAT (R. Fitch Software (Version 2007), Bad Krozingen, Germany) was used. The difference between the individual groups was checked using a *t*-test and ANOVA. The calculation of sensitivity, specificity, positive predictive value, and negative predictive value was carried out using a four-field table. We performed a Receiver Operating Characteristic (ROC) analysis and calculated the area under the curve (AUC) to evaluate how effectively the LC urine/GFR quotient, serum and urine light chains, and eGFR can differentiate between CN and other conditions. The differences among the four groups were compared using a Kruskal–Wallis Test. If significant differences were found, they were further examined using a post hoc Dunn’s Test, and *p*-values were adjusted for multiple comparisons using the Benjamini–Hochberg procedure.

## 3. Results

Between January 2019 and September 2023, a total of 67 patients (n = 24 female, n = 43 male) were included. Median age was 72 years (range: 38 to 90 years). The patients had a mean creatinine of 3.24 mg/dL (range: 0.6 mg/dL–13.0 mg/dL). In the case of proteinuria, the mean value was 2958 mg/24 h (range: 100 mg/24 h–18,887 mg/24 h).

Kidney biopsy revealed CN in 18 of the 67 cases. All histological findings can be found in Table 1.

In 17 of the 18 cases, CN occurred at the time of initial diagnosis. In the remaining case, CN occurred 20 months after the initial diagnosis of MM. At the time of initial diagnosis, a kidney biopsy was not performed due to normal kidney values and low proteinuria, as there was no suspicion of kidney involvement. At recurrence, the paraprotein was the same as at initial diagnosis (IgG kappa). Initial therapy in this patient consisted of induction therapy with bortezomib, cyclophosphamide, and dexamethasone, followed by autologous stem cell transplantation. The kidney biopsy was then carried out in cases of kidney failure requiring dialysis and previously normal kidney values. Here, progression with CN was diagnosed as the cause of kidney failure. Despite the rapid initiation of therapy with carfilzomib and dexamethasone as well as plasmapheresis, the patient remained on dialysis until his death. In three of the thirteen patients with AL amyloidosis, kidney failure appeared during the course of the disease (on average 117.4 months after initial diagnosis). Two out of three patients did not receive any treatment before because there was a misdiagnosis of MGUS. The initial workup did not include the determination of proteinuria in these patients. The third patient received induction with bortezomib and dexamethasone, followed by autologous stem cell transplantation before kidney biopsy. The kidney involvement then became apparent as part of the recurrence. One patient experienced progression after 76.4 months, with evidence of MIDD in the kidney biopsy. The patient had previously received bortezomib-based therapy, followed by an autologous transplant.

Patients with CN had significantly higher creatinine levels than patients with other histological findings (6.85 mg/dL vs. 1.92 mg/dL; *p* < 0.0001). In the case of eGFR, patients with CN had significantly lower values than patients with OKD (mean 9.93 mL/min/1.73 m^2^ vs. 38.6 mL/min/1.73 m^2^; *p* < 0.0001). There was no significant difference in the level of proteinuria between CN and ORD (mean 3929 mg/24 h vs. 2565 mg/24 h; *p* = 0.25). A more detailed breakdown of the results can be found in Table 2.

We analyzed 38 patients with kappa LC and 29 with lambda LC. Immunofixation showed complete immunoglobulin in 49 patients and only light chains in 18. In patients with CN, a kappa light chain was found in the serum in 11 of 18 cases, and only 7 cases had a lambda light chain.

**Figure 1:**
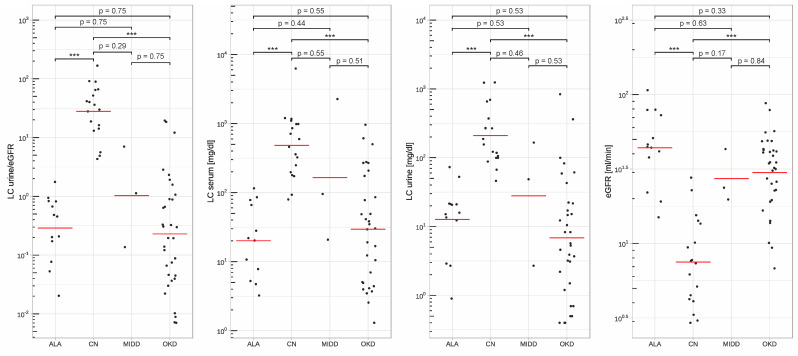
Distribution of the results of the LCurine/eGFR ratio, involved LC concentration in serum and urine and eGFR. Abbreviations: ALA = AL amyloidosis; CN = Cast nephropathy; LC = Light chain; MIDD = Monoclonal immunoglobulin deposition disease; OKD = Other kidney disease. *** *p* < 0.05.

All patients with CN were correctly predicted by the LCurine/eGFR quotient (sensitivity of 100%, 18 out of 18) for CN. In seven cases the LCurine/eGFR quotient was false positive (5x LC kappa, 2x LC lambda). In five of seven cases, a proximal light chain tubulopathy (3x kappa LC, 2x lambda LC) was histologically evident. In two patients, the histological result revealed nephroangiosclerosis and thrombotic microangiopathy, respectively. The specificity of our quotient for all patients was 85.7%. This results in a positive predictive value (PPV) of 0.72. See also Table 3. A graphical representation of the distribution of our results can be found in Figure 1. Figure 1 graphically shows the peculiarity of the particularly low eGFR in CN compared to other kidney diseases and at the same time, a high concentration of LC in the urine.

**Figure 2:**
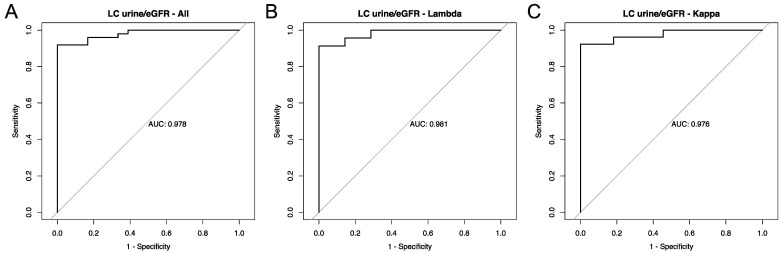
ROC analysis for the LCurine/eGFR ratio. (**A**): All patients; (**B**): Lambda-LC; (**C**): Kappa LC. Abbreviations: AUC = area under the curve.

When analyzing separately for kappa and lambda LC, the sensitivity was also 100% in both groups. The specificity was slightly higher in patients with a lambda light chain than in patients with a kappa light chain (90.9% vs. 81.5%). The PPV for the lambda LC was 0.778, and for the LC kappa, a PPV of 0.688 was calculated. To check the effectiveness and accuracy of our quotient in relation to the separation between CN and OKD, we performed a ROC analysis and calculated the AUC for the entire group as well as for the kappa-LC and the lambda-LC separately. These are shown in Figure 2.

## 4. Discussion

In line with our initial publication, we were able to show that the LCurine/eGFR ratio can be used to discriminate between CN and other kidney diseases in patients with MM and kidney failure [1]. The sole use of values already collected as part of the routine diagnosis of multiple myeloma makes rapid use possible without the determination of additional laboratory values. Above all, the high negative predictive value of the quotient of 100% makes it possible to rule out CN, which is valuable for everyday clinical practice. While the sensitivity for kappa and lambda was identical at 100%, the specificity showed a better result for patients with a lambda light chain. This is also in accordance with our initial publication. The main reason for false positive results (five of seven) are patients with unspecific tubulointerstitial damage due to high light chain concentrations in the serum and the resulting high LCurine concentration. In patients with unspecific tubulointerstitial damage, the LCurine/eGFR ratio was on average 6.7 (standard deviation 7.5). Unspecific tubulointerstitial damage is mostly caused by kappa light chains, which explains the lower specificity of the LCurine/eGFR ratio in patients with kappa light chain disease [11]. There are currently no specific treatment recommendations for patients with multiple myeloma and evidence of nonspecific tubulointerstitial damage on kidney biopsy. There are also no data as to whether patients with nonspecific tubulointerstitial damage benefit from plasmapheresis or high-cutoff dialysis HC-HD.

As shown above, a distinction between CN and ORD is also possible based on a significantly lower eGFR in patients with CN. However, as already shown in our initial publication, this is not a reliable criterion, as it does not allow a distinction to be made between CN and LCDD. It was not possible to establish a cutoff value for eGFR to distinguish between CN and ORD. This means that this is not an adequate tool in everyday clinical practice. Our quotient takes advantage of the fact that with CN, kidney function (measured as eGFR) drops significantly more than with ALA, and at the same time the LCurine concentration is higher than in patients with MIDD [1]. This leads to high sensitivity and specificity. However, differentiation within the ORD group is not possible based on the quotient.

The only other non-invasive method to diagnose CN is to measure urinary albumin excretion. Albumin is mainly found in the urine in glomerular damage. Since CN is mainly tubular damage, albumin accounts for only a small proportion of protein excretion in CN. With a limit of less than or equal to 10% albumin in proteinuria, the method shows almost the same values for sensitivity and specificity as our method [12,13]. While our method has better sensitivity (100% vs. 98%) and negative predictive value (NPV) (1.0 vs. 0.99), the specificity (86% vs. 94%) and positive predictive value (PPV) (0.72 vs. 0.75) described for the determination of the albumin fraction is better. As can be seen from the values, these are only small differences. In addition, the urine albumin fraction method is not able to distinguish CN from other tubular damage, e.g., that caused by medication.

Our quotient also has limitations. It goes without saying that it is not possible to determine our quotient in patients with anuria. Here, kidney biopsy remains the only way to rule out or prove CN. A second problem lies in the technical implementation of urine collection. To do this, urine must be collected over 24 h. In practice, however, this is often difficult for patients, and can lead to relevant shifts in the collection period. Since, in most cases, patients with acute kidney failure and the question of CN are inpatients, medical and nursing staff must ensure that the procedure is carried out correctly. Another weak point of our ratio is the false positive results in patients with light chain proximal tubulopathy. This pathology also often occurs with a severe restriction of eGFR and a high concentration of LCurine. A distinction between CN and light chain proximal tubulopathy is only possible using kidney biopsy. It is unclear whether these patients benefit from plasmapheresis. A review of our ratio on a larger collective or a prospective study would be desirable in order to be able to clarify these questions.

Kidney failure means a poorer outcome in patients with MM compared to patients without kidney failure, and dialysis is a significant impairment of quality of life [14,15,16]. CN has the worst outcome in terms of kidney failure compared to other kidney diseases such as amyloidosis or MIDD. On the other hand, CN also shows the highest rate of recovery of kidney function with adequate therapy [16]. Above all, the rapid and effective reduction of the light chain concentration in the serum is decisive for this. In addition to high fluid intake (>3 L/day) and systemic therapy (corticosteroids and proteasome inhibitors), this can also include the mechanical removement of light chains, for example, with plasmapheresis [13,17]. However, the mechanical removal of light chains from plasma appears to be effective only in patients with CN. Thus, current IMWG criteria recommend mechanical removal in patients with acute kidney failure with proven CN [18]. The mechanical reduction of LC can either be performed via plasmapheresis or HC-HD. There is a very small amount of data for both these therapies. None of the three studies that examined plasmapheresis for the treatment of myeloma patients could show any difference in mortality [19,20,21]. However, two of the three studies [19,21] were able to numerically show an improvement in kidney function and a larger proportion of HD-free patients at the end of therapy. The third study, by Clark et al., showed no difference in the recovery of kidney function. However, since almost no kidney biopsies were carried out in this study, it is unclear what was actually being treated here. Since only about 60% of cases of acute kidney failure in multiple myeloma are caused by CN, it must be assumed that patients with non-CN-mediated kidney failure also received plasmapheresis. In most non-CN-mediated kidney failures, the level of paraprotein plays only a very minor role, so no benefit from plasma separation can be assumed here [10].

There are only two studies available for extracorporeal LC removal using HC-HD. The so called MYRE-Trial showed a significant improvement in kidney function after 6 and 12 months when using HC-HD. However, this did not affect the overall survival (OS) [22]. In the EuLITE study, conducted in the United Kingdom, there was no difference in kidney function but significantly worse OS (hazard ratio—2.63, *p* = 0.03) when using HC-HD [23]. The EuLITE study also demonstrated a lower hematologic response at 6 months than the MYRE trial. This was reflected in lower “very good partial remission” (VGPR) (23 vs. 32%) and “complete remission (CR) (14% vs. 30%). The higher mortality in the HC-HD group was due to an increased rate of infectious complications (especially pulmonary infections). The value of HC-HD for the treatment of myeloma-induced kidney failure is currently unclear. Further large phase 3 studies are necessary in order to be able to make statements about possible use.

The only way to prove CN is a kidney biopsy. As an invasive procedure, however, there is a risk of bleeding. This is further increased by high LC concentrations in the serum, so that in some cases of MM-induced kidney failure, a kidney biopsy cannot be performed [24]. Due to the high negative predictive value of the LCurine/eGFR quotient, the group of patients who do not benefit from the mechanical removal of light chains can be determined non-invasively without exposing them to the risk of bleeding. The lower specificity of the quotient does not allow a reliable diagnosis of CN in the case of a positive finding. The lower specificity in the group of patients with a kappa-LC is due to the fact that, although in the group of patients with CN the kappa/lambda ratio is almost equally distributed (10 kappa-LC vs. 8 lambda-LC), there are significantly more patients with a kappa-LC in the overall group (38 kappa-LC against 29 lambda-LC). This results in a greater confounding factor being present in the group of patients with kappa-LC.

In summary, our data show that the LCurine/eGFR quotient is a valuable, non-invasive tool to rule out CN in patients with diagnosed MM and acute kidney failure. Its simple calculation with values already determined as part of the routine (concentration of light chains in the urine as well as eGFR and proteinuria) makes it a valuable tool. This allows further steps in diagnostics and therapy to be initiated quickly and easily, saving the patient time and unnecessary diagnostics.

## 5. Conclusions

In everyday clinical practice, our quotient shows a sensitivity of 100% and a specificity of 85.7% for the presence of cast nephropathy in patients with multiple myeloma and kidney failure. In addition to the fact that our quotient is quicker and easier to perform than a kidney biopsy, it is also significantly more cost-effective (in Germany, the pathological work-up alone costs around EUR 300, plus the costs of carrying it out) than a kidney biopsy.

## Figures and Tables

**Table 1:** Histological results of the kidney biopsy (n = 67).

Histological Findings	Number (n)
Cast nephropathy	18
Light chain proximal tubulopathy	9
Membranoproliferative glomerulonephritis	1
Intrarenal myeloma/lymphoma	2/1
AL amyloidosis	13
MIDD	3
Nephroangiosclerosis	6
IgA glomerulonephritis	2
Thrombotic microangiopathy	2
Membranous glomerulonephritis	3
C3 glomerulonephritis	1
Acute tubular damage	3
Focal segmental glomerulosclerosis	1
Interstitial nephritis	1
Diabetic nephropathy	1

Abbreviations: MIDD = monoclonal immunoglobulin deposition disease.

**Table 2:** Breakdown of the results of eGFR, LC urine, LC serum, and LC urine/eGFR ratios by histology. Median (interquartile range).

	CN (n = 18)	ALA (n = 13)	MIDD (n = 3)	Others (n = 33)	*p*-Value
**eGFR (mL/min**/**1.73 m^2^)**	7.5 (8.4)	44.4 (34.8)	23.7 (11.6)	34.9 (19.9)	CN vs. ALA *p* < 0.05 CN vs. Others *p* < 0.05
**Involved FLC serum (mg/dL)**	598.0 (789.0)	21.1 (61.8)	95.5 (1124.6)	29.3 (125.0)	CN vs. ALA *p* < 0.05 CN vs. Others *p* < 0.05
**Involved LC urine (mg/dL)**	171.5 (244.8)	15.9 (8.9)	48.6 (81.7)	5.7 (20.8)	CN vs. ALA *p* < 0.05 CN vs. Others *p* < 0.05
**LCurine/eGFR Quotient**	33.1 (46.9)	0.5 (0.6)	1.1 (3.4)	0.2 (0.9)	CN vs. ALA *p* < 0.05 CN vs. Others *p* < 0.05 CN vs. MIDD *p* < 0.05

Abbreviations: ALA = AL amyloidosis; CN = Cast nephropathy; FLC = Free light chain; MIDD = Monoclonal immunoglobulin deposition disease.

**Table 3:** Results.

**All Patients**	**CN Yes (n = 18)**	**CN No (n = 49)**	**PPV/NPV**
Quotient positive	18	7	0.72
Quotient negative	0	42	1
Sensitivity/specificity	1	0.857	
**Lambda LC**	**CN Yes (n = 7)**	**CN No (n = 22)**	**PPV/NPV**
Quotient positive	7	2	0.778
Quotient negative	0	20	1
Sensitivity/specificity	1	0.909	
**Kappa LC**	**CN Yes (n = 11)**	**CN No (n = 27)**	**PPV/NPV**
Quotient positive	11	5	0.688
Quotient negative	0	22	1
Sensitivity/specificity	1	0.815	

Abbreviations: CN = Cast nephropathy; LC = Light chain; NPV = Negative predictive value; PPV = Positive predictive value.

## Data Availability

The raw data supporting the conclusions of this article will be made available by the authors on request.

## References

[1] Bergner R., Hoffmann M., Uppenkamp M., Paschka P., Klank D. (2021). The Urine Light Chain/Glomerular Filtration Rate (GFR) Quotient Shows a High Sensitivity and Specificity to Detect Cast Nephropathy in Monoclonal Light Chain Disease. Eur. J. Haematol..

[2] Yadav P., Cockwell P., Cook M., Pinney J., Giles H., Aung Y.S., Cairns D., Owen R.G., Davies F.E., Jackson G.H. (2018). Serum Free Light Chain Levels and Renal Function at Diagnosis in Patients with Multiple Myeloma. BMC Nephrol..

[3] Knudsen L.M., Hippe E., Hjorth M., Holmberg E., Westin J. (1994). Renal Function in Newly Diagnosed Multiple Myeloma—A Demographic Study of 1353 Patients. Eur. J. Haematol..

[4] Rajkumar S.V. (2016). Updated Diagnostic Criteria and Staging System for Multiple Myeloma. Am. Soc. Clin. Oncol. Educ. B.

[5] Knudsen L.M., Hjorth M., Hippe E. (2000). Renal Failure in Multiple Myeloma: Reversibility and Impact on the Prognosis. Eur. J. Haematol..

[6] Ng K.P., Fallouh B., Baharani J. (2012). Short and Long-Term Outcome of Patients with Severe Acute Kidney Injury Requiring Renal Replacement Therapy. Qjm.

[7] Laing A.A., Geddes C., Soutar R. (2015). Renal Impairment at Presentation in Multiple Myeloma Continues to Be Associated with Poor Survival. Br. J. Haematol..

[8] Rüsing L.Z., Kozakowski N., Jeryczynski G., Vospernik L., Riedl J., Reiter T., Gisslinger H., Agis H., Krauth M.T. (2024). Renal Outcome in Multiple Myeloma Patients with Cast Nephropathy: A Retrospective Analysis of Potential Predictive Values on Clinical and Renal Outcome. Hematology.

[9] Haynes R.J., Read S., Collins G.P., Darby S.C., Winearls C.G., Ludwig H., Adam Z., Hajek R., Greil R., Tóthová E. (2010). Light Chain-Induced Acute Renal Failure Can Be Reversed by Bortezomib-Doxorubicin-Dexamethasone in Multiple Myeloma: Results of a Phase II Study. Nephrol. Dial. Transplant..

[10] Klank D., Hoffmann M., Porubsky S., Bergner R. (2022). Histological Findings in Kidney Biopsies of Patients with Monoclonal Gammopathy—Always a Surprise. Diagnostics.

[11] Fogo A.B., Lusco M.A., Najafian B., Alpers C.E. (2016). AJKD Atlas of Renal Pathology: Light Chain Proximal Tubulopathy. Am. J. Kidney Dis..

[12] Leung N., Gertz M., Kyle R.A., Fervenza F.C., Irazabal M.V., Eirin A., Kumar S., Cha S.S., Rajkumar S.V., Lacy M.Q. (2012). Urinary Albumin Excretion Patterns of Patients with Cast Nephropathy and Other Monoclonal Gammopathy-Related Kidney Diseases. Clin. J. Am. Soc. Nephrol..

[13] Leung N., Rajkumar S.V. (2023). Multiple Myeloma with Acute Light Chain Cast Nephropathy. Blood Cancer J..

[14] Montseny J.J., Kleinknecht D., Meyrier A., Vanhille P., Simon P., Pruna A., Eladari D. (1998). Long-Term Outcome According to Renal Histological Lesions in 118 Patients with Monoclonal Gammopathies. Nephrol. Dial. Transplant..

[15] Nasr S.H., Valeri A.M., Sethi S., Fidler M.E., Cornell L.D., Gertz M.A., Lacy M., Dispenzieri A., Rajkumar S.V., Kyle R.A. (2012). Clinicopathologic Correlations in Multiple Myeloma: A Case Series of 190 Patients with Kidney Biopsies. Am. J. Kidney Dis..

[16] Decourt A., Gondouin B., Delaroziere J.C., Brunet P., Salleé M., Burtey S., Dussol B., Ivanov V., Costello R., Couchoud C. (2016). Trends in Survival and Renal Recovery in Patients with Multiple Myeloma or Light-Chain Amyloidosis on Chronic Dialysis. Clin. J. Am. Soc. Nephrol..

[17] Bridoux F., Leung N., Belmouaz M., Royal V., Ronco P., Nasr S.H., Fermand J.P. (2021). Management of Acute Kidney Injury in Symptomatic Multiple Myeloma. Kidney Int..

[18] Dimopoulos M.A., Sonneveld P., Leung N., Merlini G., Ludwig H., Kastritis E., Goldschmidt H., Joshua D., Orlowski R.Z., Powles R. (2016). International Myeloma Working Group Recommendations for the Diagnosis and Management of Myeloma-Related Renal Impairment. J. Clin. Oncol..

[19] Zucchelli P., Pasquali S., Cagnoli L., Ferrari G. (1988). Controlled Plasma Exchange Trial in Acute Renal Failure Due to Multiple Myeloma. Kidney Int..

[20] Clark W.F., Stewart A.K., Rock G.A., Sternbach M., Sutton D.M., Barrett B.J., Heidenheim A.P., Garg A.X., Churchill D.N. (2005). Plasma Exchange When Myeloma Presents as Acute Renal Failure: A Randomized, Controlled Trial. Ann. Intern. Med..

[21] Johnson W.J., Kyle R.A., Pineda A.A., O’Brien P.C., Holley K.E. (1990). Treatment of Renal Failure Associated with Multiple Myeloma. Plasmapheresis, Hemodialysis, and Chemotherapy. Arch. Intern. Med..

[22] Bridoux F., Carron P.-L., Pegourie B., Alamartine E., Augeul-Meunier K., Karras A., Joly B., Peraldi M.-N., Arnulf B., Vigneau C. (2017). Effect of High-Cutoff Hemodialysis vs. Conventional Hemodialysis on Hemodialysis Independence among Patients with Myeloma Cast Nephropathy: A Randomized Clinical Trial. JAMA.

[23] Hutchison C.A., Cockwell P., Moroz V., Bradwell A.R., Fifer L., Gillmore J.D., Jesky M.D., Storr M., Wessels J., Winearls C.G. (2019). High Cutoff versus High-Flux Haemodialysis for Myeloma Cast Nephropathy in Patients Receiving Bortezomib-Based Chemotherapy (EuLITE): A Phase 2 Randomised Controlled Trial. Lancet. Haematol..

[24] Hinterleitner C., Pecher A.C., Kreißelmeier K.P., Budde U., Kanz L., Kopp H.G., Jaschonek K. (2020). Disease Progression and Defects in Primary Hemostasis as Major Cause of Bleeding in Multiple Myeloma. Eur. J. Haematol..

